# The Role of Auxin-Ethylene Crosstalk in Orchestrating Primary Root Elongation in Sugar Beet

**DOI:** 10.3389/fpls.2017.00444

**Published:** 2017-03-30

**Authors:** Willem Abts, Bert Vandenbussche, Maurice P. De Proft, Bram Van de Poel

**Affiliations:** ^1^Division of Crop Biotechnics, Department of Biosystems, University of LeuvenLeuven, Belgium; ^2^SESVanderHave N.V.Tienen, Belgium

**Keywords:** auxin, ethylene, sugar beet, root elongation, crosstalk

## Abstract

It is well-established in *Arabidopsis* and other species that ethylene inhibits root elongation through the action of auxin. In sugar beet (*Beta vulgaris* L.) ethylene promotes root elongation in a concentration dependent manner. However, the crosstalk between ethylene and auxin remains unknown during sugar beet seedling development. Our experiments have shown that exogenously applied auxin (indole-3-acetic acid; IAA) also stimulates root elongation. We also show that auxin promotes ethylene biosynthesis leading to longer roots. We have further demonstrated that the auxin treatment stimulates ethylene production by redirecting the pool of available 1-aminocyclopropane-1-carboxylic acid (ACC) toward ethylene instead of malonyl-ACC (MACC) resulting in a prolonged period of high rates of ethylene production and subsequently a longer root. On the other hand we have also shown that endogenous IAA levels were not affected by an ACC treatment during germination. All together our findings suggest that the general model for auxin-ethylene crosstalk during early root development, where ethylene controls auxin biosynthesis and transport, does not occur in sugar beet. On the contrary, we have shown that the opposite, where auxin stimulates ethylene biosynthesis, is true for sugar beet root development.

## Introduction

Sugar beet (*Beta vulgaris* L.) is a root crop which is primarily cultivated for extracting sugars from its tap root. The initial root development phase immediately after germination is a crucial process that primes the seedling for a steady development and is important for the further outgrowth of the tap root which ultimately determine sugar yield. Currently, little is known about the hormonal regulation of sugar beet root development.

In *Arabidopsis thaliana* multiple studies have shown that root elongation during early root development is inhibited by the gaseous plant growth regulator ethylene (Ruzicka et al., 2007; Stepanova et al., 2007; Swarup et al., 2007; Markakis et al., 2012). However, Pierik et al. (2006) have proposed a biphasic ethylene response model where ethylene has both an inhibitory and stimulatory effect on root elongation depending on the ethylene concentration and the species. Recently we have shown that the early root growth in sugar beet also shows a biphasic ethylene response (Abts et al., 2014). Application of low concentrations of the ethylene precursor 1-aminocyclopropane-1-carboxylic acid (ACC) stimulates root growth while high concentrations inhibit root growth (Abts et al., 2014). It is also known that auxin can inhibit root elongation in many species (e.g., *Arabidopsis, Brassica*, maize, pea…; Eliasson et al., 1989; Rahman et al., 2007; Ruzicka et al., 2007; Stepanova et al., 2007; Swarup et al., 2007; Alarcón et al., 2012; Polit et al., 2014). In contradiction, it was also previously shown in *Arabidopsis* that low auxin levels could stimulate root elongation (Evans et al., 1994) which might suggest that auxins can also exert a biphasic response in root growth. However, the auxin response during early root growth of sugar beet remains elusive.

The regulation of root elongation is often the result of a complex interaction between ethylene and auxin (reviewed by Benková and Hejátko, 2009; Muday et al., 2012; Van de Poel et al., 2015; Hu et al., 2017). Studies in *Arabidopsis* have shown that ethylene stimulates auxin biosynthesis and upregulates the transcription of several auxin transporters (e.g., *PIN1, PIN2, AUX1*; Ruzicka et al., 2007; Stepanova et al., 2007; Swarup et al., 2007). The ethylene-induced auxin production is localized in the root tip (Swarup et al., 2007) and the auxin signal is subsequently redistributed by polar auxin transport toward the elongation zone. This results in an auxin accumulation in the elongation zone and leads to an inhibited cell elongation (Ruzicka et al., 2007). Inhibition of auxin transport using auxin transport mutants (e.g., *pin2* and *aux1*) results in an ethylene insensitive root growth due to the lack of crosstalk possibilities (Ruzicka et al., 2007). Another possible point of auxin-ethylene crosstalk is the enzyme VAS1 which regulates both auxin and ethylene production (Zheng et al., 2013; Pieck et al., 2015).

The reciprocal regulation in which auxin controls ethylene biosynthesis during root development is also well-described (reviewed by Benková and Hejátko, 2009; Muday et al., 2012). Application of the auxin indole-3-acetic acid (IAA) induces the expression and enzyme activity of both ACC-synthase (ACS) and ACC-oxidase (ACO) in roots of both pea and *Arabidopisis* (Peck and Kende, 1995, 1998; Tsuchisaka and Theologis, 2004; Stepanova et al., 2007). The complex crosstalk between ethylene and auxin also occurs during the regulation of root gravitropism (Lee et al., 1990), root hair initiation and elongation (Tanimoto et al., 1995; Pitts et al., 1998; Rahman et al., 2002), hypocotyl growth (Collett et al., 2000) and apical hook formation (Lehman et al., 1996). The formation of malonyl-ACC (MACC) by ACC-N-malonyltransferase (Martin and Saftner, 1995), as a mechanism to control the pool of ACC and subsequently ethylene production levels, is often neglected in ethylene and hormonal crosstalk studies (Van de Poel and Van Der Straeten, 2014).

In a previous study we have shown that ethylene regulates root elongation during sugar beet germination in a dose-dependent manner (Abts et al., 2014). However, the involvement of auxin during early root growth in sugar beet seedlings remains unresolved. In order to study the relation between auxin and ethylene during early root growth of sugar beet seedlings, kinetic germination assays were set up, the ethylene biosynthesis pathway was studied and endogenous IAA levels in sugar beet fruits and seedlings were quantified during root growth. Our results show that IAA stimulates ethylene production, resulting in a prolonged period of ethylene exposure which leads to longer roots. We further show that this IAA-stimulated ethylene production is likely achieved by inhibiting the conversion of ACC to MACC. We therefore propose that the auxin-stimulated ethylene production is responsible for the promotion of root elongation in sugar beet seedlings.

## Materials and methods

### Plant material, morphology, and seed germination

Diploid monogerm sugar beet (*B. vulgaris* L.) fruits consisting of a true seed surrounded by pericarp, all originated from one seed lot (LZD-2386, SESVanderHave N.V.). The seed lot was produced in 2010 (France, Nérac) and fruits were processed to meet commercial standards. The fruits were stored at room temperature and 35% relative humidity until further use.

For germination experiments, independent triplicates of 100 fruits were incubated in darkness at 20°C in polystyrene Petri dishes (90 mm), containing one layer of moist filter paper (Whatman No 1; 3 mL deionized water). Each Petri dish contained 25 fruits. Germination was counted at specific time intervals. Radicle protrusion of both seed coats was used as criterion for germination. Where indicated indole-3-acetic acid (IAA; Acros), 1-aminocyclopropane-1-carboxylic acid (ACC; Acros), α-(p-chlorophenoxy)isobutyric acid (PCIB, Sigma-Aldrich), or silver thiosulfate (STS; Sigma-Aldrich) was added to the imbibition medium. Silver thiosulfate was prepared as described by Reid et al. (1980). PCIB was dissolved in dimethyl sulfoxide (DMSO, Sigma-Aldrich) and diluted to the appropriate concentration. The final concentration of DMSO was kept below 0.1% for all treatments.

For root length measurements, at least 40 seedlings were used. Total root length was measured using a stereo microscope (Olympus SZX9) equipped with a digital camera (Olympus, Colorview II) and a video image analysis software (Olympus, Cell B).

### Ethylene and *In vivo* ACO enzyme activity measurements

Ethylene production was measured using gas chromatography as described by Abts et al. (2013). Briefly, 20 seedlings were incubated in glass flasks (10 mL) at 20°C always in five replicates. After 1 h incubation 1 mL headspace was sampled and analyzed for ethylene content. The accumulated ethylene in the headspace was measured using gas chromatography (Shimadzu GC-2014) equipped with a packed column and a flame ionization detector. The injector, the column and the detector had temperatures of 150, 90, and 250°C, respectively.

*In vivo* ACO enzyme activity was measured as described by Abts et al. (2014). Briefly, the *in vivo* ACO enzyme activity was determined by measuring the maximal ethylene production. At specific time intervals the sugar beet seedlings were carefully removed from each Petri dish and incubated for 3 h in another Petri dish containing 3 mL of a saturating 1 mM ACC solution. Subsequently the seedlings were incubated for 30 min in a gas-tight 10 mL glass flask and ethylene content in the headspace was measured as described above.

### ACC, MACC, and IAA quantification

ACC was extracted and quantified using the Lizada and Yang (1979) method optimized by Bulens et al. (2011). Briefly, ACC and MACC was extracted from 0.5 g of frozen and crushed seedling tissue with 1 mL 5% sulfosalicylic acid (Sigma) for 30 min at 4°C. Subsequently the sample was centrifuged for 10 min at 5,000 × g at 4°C. The amount of ACC extract was quantified by converting it to ethylene using a saturated NaOH:NaOCl (5%) mixture and HgCl_2_ (10 mM). The reaction mixture was incubated for 4 min on ice, vortexed and subsequently a 1 mL headspace sample was analyzed with the GC for ethylene content. The reaction efficiency was determined in a second analyses of the same sample, by spiking with 10 μL 10 μM ACC. MACC was converted into ACC by an acid hydrolysis according to Hoffman et al. (1982) also updated by Bulens et al. (2011). Briefly, 100 μL of the ACC extract was hydrolysed for 4 h at 100°C using 6 M HCl. The hydrolysed sample was neutralized with 6M NaOH, centrifuged for 5 min at 13,000 × g and the supernatants was collected and analyzed for total ACC content (hydrolysed ACC + free ACC) as described above.

IAA was extracted from entire sugar beet seedlings based on the extraction procedure for auxins described earlier by Prinsen et al. (2000). Homogenized plant material was extracted in 80% methanol (10 mL/g fresh weight) and extracted overnight. A stable isotope-labeled IAA ([^13^C_6_]-IAA, 50 pmol, CLM-1896-PK, Cambridge Isotope Laboratories Inc., Andover, Massachusetts, USA) was added as internal standard. After centrifugation (20,000 × g for 15 min at 4°C) the supernatant was passed over a C18 cartridge (500 mg) to retain pigments. The effluent was diluted to 50% methanol and concentrated on a DEAE-Sephadex anion exchange column (2 mL) for the analysis of free IAA, which was retained on the DEAE. The DEAE cartridge was eluted with 10 mL 6% formic acid and free IAA was subsequently concentrated on a C18 cartridge. This C18 cartridge was eluted with 2 × 0.5 mL diethylether. The ether was evaporated under *vacuo* and the sample was suspended in acidified methanol for methylation with diazomethane. After methylation, the samples were dried under a nitrogen stream and samples were further dissolved in 50 μL 10% MeOH (Prinsen et al., 2000).

IAA was analyzed by UPLC-MS/MS (Acquity TQD, Waters, Manchester, UK; 6 μL injection by partial loop, column temperature 30°C, solvent gradient 0–2 min: 95/5; 10% MeOH in 1 mM NH_4_OAc/MeOH; 2–4 min linear gradient until 10/90 10% MeOH in 1 mM NH_4_OAc/MeOH; 4–6 min, isocratic 10/90 10% MeOH in 1 mM NH_4_OAc/MeOH). MS conditions were set at: polarity MS ES(+), capillary 2 kV, cone 20 V, collision energy 20 eV, source temperature 120°C, desolvation temperature 450°C, cone gas flow 50 L/h, desolvation gas flow 750 L/h, and collision gas flow 0.19 mL/h. The diagnostic ions used for quantification are 190 > 130 m/z for Me-IAA and 196 > 136 m/z for Me-[^13^C_6_]-IAA (dwell time 0.02 s). Methanol and water used for MS are UPLC grade.

### RNA extraction and RT-qPCR

Total RNA was extracted using the RNeasy Plus Mini Kit (Qiagen) from 0.1 g frozen crushed sugar beet seedlings. The manufacturer's protocol was used. Briefly each sample was homogenized in 600 μL of Buffer RLT Plus supplemented with 10 μL β-mercaptoethanol. A 1% agarose gel stained with ethidium bromide was used to check RNA integrity. RNA purity was determined by the 260/280 and 260/230 nm ratio. Both the RNA content as well as the RNA purity were measured with the NanoDrop 2000 (Thermo Scientific). The QuantiTect Reverse Transcription Kit (Qiagen) was used to reverse transcribe 1 μg of the total RNA into cDNA according to the manufacturer's protocol. Samples were stored at −80°C until further use.

The expression profiles of all known isoforms of *ACS* and *ACO* (Dohm et al., 2014; Table 1) in sugar beet were determined during early seedling growth. Quantification was obtained via real-time quantitative PCR (RT-qPCR) on a Rotor-Gene Q cycler (Qiagen) for 45 cycles. The RT-qPCR reaction consisted of a forward and reverse primer (3.75 μM), RT-template, water, and Absolute QPCR SYBR Green mix (Abgene Limited, Epsom, UK). The used primers and their properties are listed in Table 1. Primers were designed with Primer3 software (Rozen and Skaletsky, 2000).

**Table 1:** **List of used primers and their properties for the quantification of mRNA abundance**.

**Gene code**	**Accession**	**Forward-primer (5′->3′)**	**Reverse-primer (5′->3′)**
*ACS1*	Bv_27160_idme.t1	ATGAGCCAACAAAGGAAAGTG	TGACAAGATGAACCAGGAGAGA
*ACS2*	Bv5_094750_hwtr.t1	TCGCATAGTAATGAGTGGTGGA	CAGGATAGTAGGGTGTGGGAAC
*ACS3*	Bv1_004170_gcto.t1	CAGGGTGGTTTAGGGTTTGTT	TGGTCTTTCCTCCATTTTTCC
*ACS4*	Bv6_125280_uenw.t1	CCAACACCAACAACAACAACA	TCTCGTATTCTTCCCAACCAA
*ACO1*	Bv_37910_nwgk.t1	GAGCTGATGTGTGAAAACCTTG	CGCTACCTTTGTTCCTACTGCT
*ACO2*	Bv9_207650_gdtf.t1	TGGGGTTTCTTTGAGTTGATG	CACTTCTTGTAATGCTCCTTTGTG
*ACO3*	Bv4_084910_gucu.t1	TAGAGGGCAAGGATGACAAAA	CAAAAGAGCGGCAAAACTATC
*ACO4*	Bv3_051500_mqsu.t1	CCTCAACCTGATGCTTTTGTT	CTGCTCTGTGCCATACACTCTT
*ACO5*	Bv8_187920_wndh.t1	CTGTGGAAAGGATGACAAAAGA	TGATGGCGAAGGTAGAAAGTG
*Actin*	HQ656028	CACGAGACAACCTACAACTCCA	GCTCATACGGTCAGCAATACC
*18S rRNA*	FJ669720	GAAAGACGAACAACTGCGAAA	CATCGTTTATGGTTGAGACTAGGA

Specificity of amplification was confirmed performing a melting curve analysis after each qPCR run. The melting curve was obtained by increasing the temperature in steps of 0.5°C/s ranging from 55 to 95°C. Three biological replicates were used and normalized against the average expression of two reference genes (*Actin* and *18S rRNA*). Relative quantification was calculated by including a calibration curve in duplex in each run.

### Statistics

For the root length measurements significant differences between the different treatments and days were calculated using SAS Enterprise Guide 6 with the linear models procedure and the Tukey means comparison test set with a 95% confidence interval. All other statistical differences were analyzed with the one-way ANOVA procedure followed by a Tukey multiple comparison test using the statistical package “R” version 2.12.2. Significance level was set at 5%.

## Results

### Sugar beet root length is regulated by an interaction between auxin and ethylene

**Figure 1:**
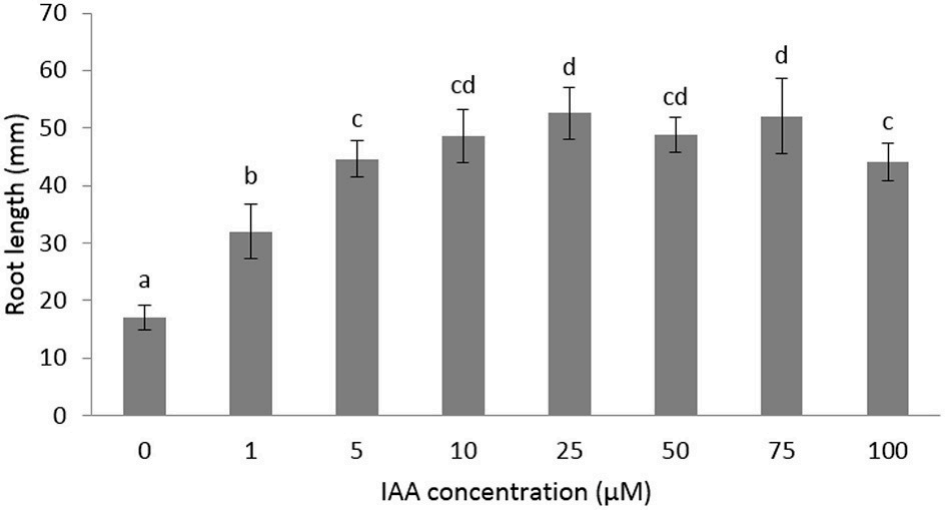
**Effects of different concentrations of indole-3-acetic acid (IAA) added to the imbibition medium on the root length of sugar beet seedlings after 6 days at 20°C in darkness**. Mean values ± *SD* are presented (*n* > 40). Levels of significance (*p* < 0.05) are indicated with the letters a–d.

The role of auxin and ethylene during root growth is quite well-established in *Arabidopsis* and some other crops (Muday et al., 2012), but is still unclear in sugar beet. Furthermore, the biphasic behavior of ethylene on root growth is species dependent (Pierik et al., 2006) and might suggest a differential crosstalk with auxin. In order to investigate the role of auxin and ethylene on early root development of sugar beet we measured the root in the presence of different imbibition media. We first tested the effect of auxin on root elongation using different IAA concentrations ranging between 1 and 100 μM (Figure 1). Root length was significantly stimulated for IAA concentrations between 1 and 10 μM. Higher concentrations (25–100 μM IAA) did not result in a stronger elongation response (Figure 1). The 10 μM IAA treatment was chosen in subsequent experiments to unravel the ethylene-auxin crosstalk in sugar beet seedlings because this concentration of IAA was the lowest IAA concentration at which a maximal root elongation response was observed.

**Figure 2:**
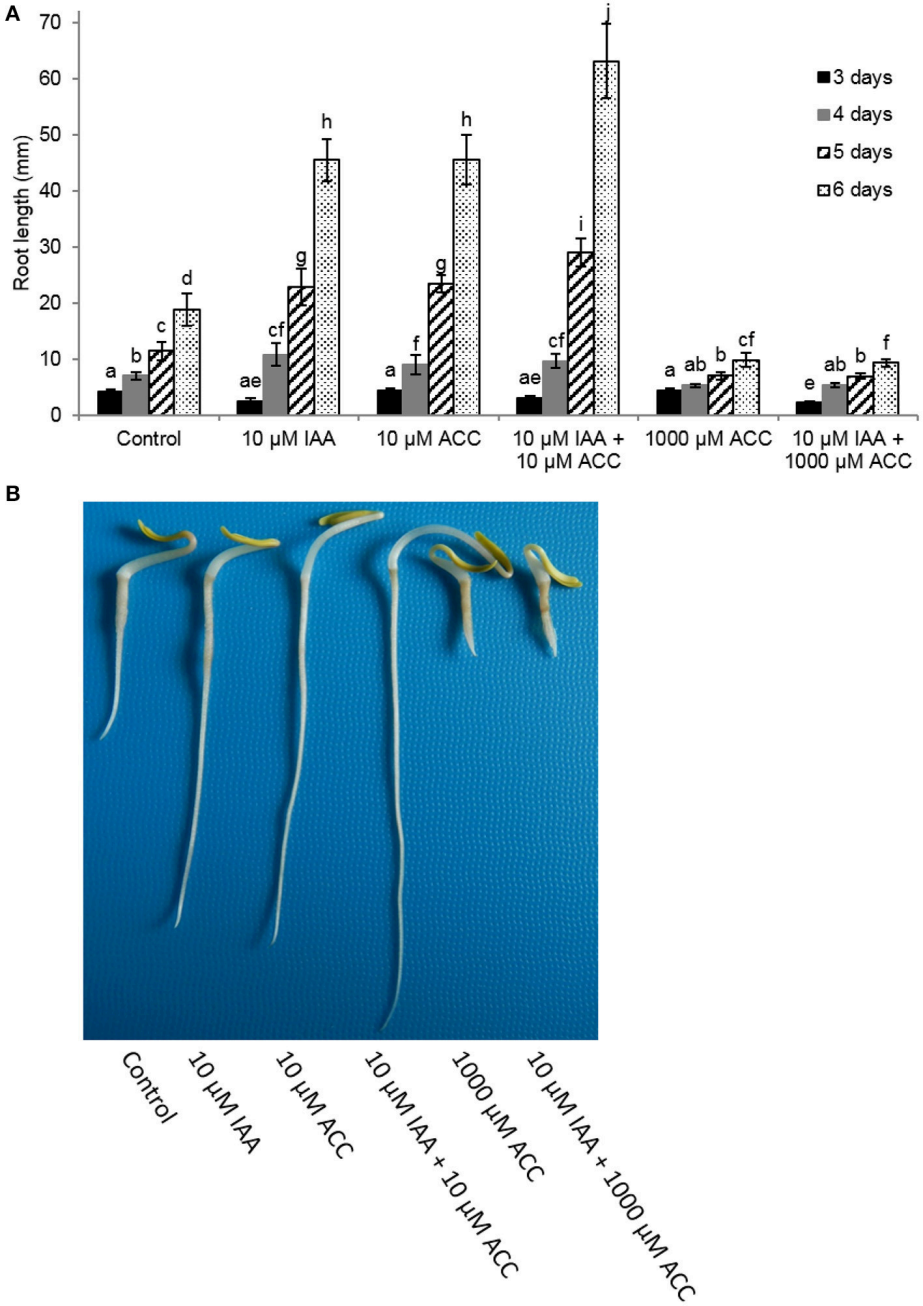
**Effect of indole-3-acetic acid (IAA) and 1-aminocyclopropane-1-carboxylic acid (ACC) added to the imbibition medium on (A)** root length (mm) of sugar beet seedlings after 3, 4, 5, and 6 days at 20°C in darkness. Mean values ± *SD* are presented (*n* > 40). A linear models procedure and the Tukey means comparison test was used to check statistical differences between the treatments and days. Levels of significance (*p* < 0.05) are indicated with the letters a–j. **(B)** Representative examples of 6 day old sugar beet seedlings incubated at 20°C in darkness in the presence of different 1-aminocyclopropane-1-carboxylic acid (ACC) and/or indole-3-acetic acid (IAA) concentrations.

Next, we measured root elongation over a 6 day period for different IAA and ACC combinations (Figure 2). All treatments stimulated root growth and the largest effects were observed 6 days after imbibition. At that time point the root length of seedlings grown on 10 μM IAA were more than double in length compared to the untreated control. The addition of 10 μM ACC to the imbibition medium also stimulated root elongation similar as the 10 μM IAA treatment. Root length was even more stimulated when a combination of 10 μM IAA + 10 μM ACC was applied indicating a cooperative action of ACC and IAA. Root elongation was inhibited when seedlings were treated with a very high ACC concentration (1 mM), which was in accordance with our previous observations (Abts et al., 2014). In the presence of the high ACC concentration of 1 mM, the addition of 10 μM IAA did not reverse the inhibition of root elongation. Both ACC treatments (10 μM and 1 mM) also stimulated ethylene production of the sugar beet seedlings yet to a different extent (Supplementary Figure 1). A small increase in ethylene production (10 μM ACC) leads to an increase in root elongation, while a large increase in ethylene production (1 mM ACC) inhibits root elongation (Supplementary Figure 1).

**Figure 3:**
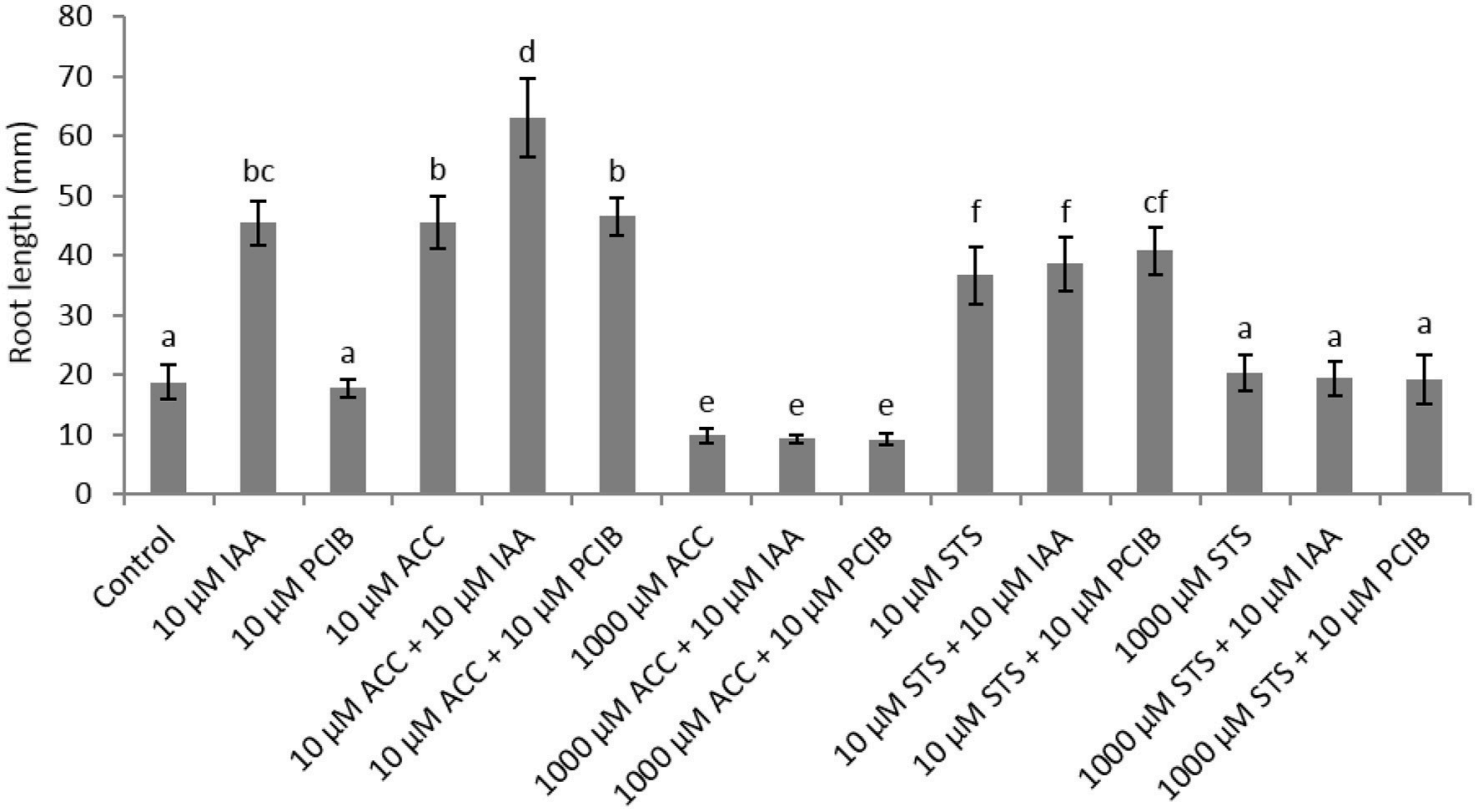
**Effect of indole-3-acetic acid (IAA), 1-aminocyclopropane-1-carboxylic acid (ACC), p-chlorophenoxyisobutyric acid (PCIB), silver thiosulphate (STS), and the combinations of these plant growth regulators on the root length (mm) of sugar beet seedlings after 6 days at 20°C in darkness**. Mean values ± *SD* are presented (*n* > 40). Levels of significance (*p* < 0.05) are indicated with the letters a–f.

In order to further investigate the role of auxin on root elongation in sugar beet we have performed germination experiments with the auxin response inhibitor α-(p-chlorophenoxy)isobutyric acid (PCIB; Oono et al., 2003) in combination with different concentrations of ACC (Figure 3). A PCIB treatment did not affect root elongation when administered in different concentrations (Supplementary Figure 2). The ethylene-induced root elongation response of seedlings was not influenced when administered 10 μM PCIB together with 10 μM ACC, indicating that the ethylene response is independent from the action of auxin. When 1,000 μM ACC was supplemented, the additive effect of 10 μM IAA or PCIB is abolished. The PCIB treatment did not influence ethylene production during sugar beet germination (Supplementary Figure 3). In order to evaluate the auxin-stimulated effect on root elongation we used the ethylene perception inhibitor silver thiosulphate (STS) in combination with 10 μM IAA. Surprisingly the 10 μM STS treatment resulted in an increased root elongation, while the 1,000 μM STS treatment did not affect root growth compared to the untreated controls. Despite this positive effect of 10 μM STS on root growth, the combined treatment with IAA (or PCIB) did not promote or inhibit root elongation (Figure 3). The increase in root length observed for the 10 μM STS treatment might be explained by the subtle but significant increase in ethylene production of the STS treatment (Supplementary Figure 4). Previous work with STS as an ethylene response inhibitor also reported a drastic increase in ethylene production in tomato fruit (Atta-Aly et al., 1987). At the end, one can question the functionality of STS to inhibit ethylene responses. Perhaps the STS treatment causes phytotoxicity or abiotic stress (silver as a heavy metal), which might trigger ethylene production.

### IAA does not affect germination rate of sugar beet

**Figure 4:**
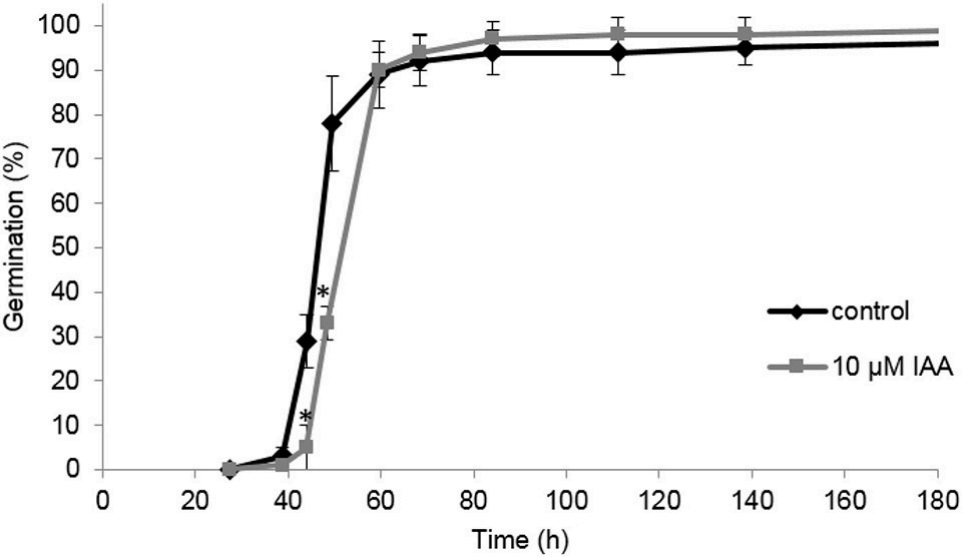
**Effect of indole-3-acetic acid (IAA) on the germination percentage of sugar beet incubated at 20°C in darkness**. Each point represents the mean of three independent replicates of 100 fruits. Mean values ± *SD* are presented. The asterisk indicates that the IAA treatment differs significantly from the corresponding controls (*p* < 0.05).

In this study we aim to unravel the effect of IAA and ethylene (via ACC supplementation) on root elongation. However, a possible delay or promotion of germination might mask a root elongation response, complicating the interpretation of the effect of IAA and ACC. Consequently, it is possible that differences in root length are (partially) caused by an altered germination rate. It was shown previously that ACC had no effect on the germination rate of sugar beet fruits (Abts et al., 2013). Interestingly, IAA delayed germination for just a few hours (Figure 4). The time to reach 50% germination (t_50_) was 46 h for the control, compared to 52 h for the IAA treatment. The time to reach 90% germination (t_90_) was not influenced by IAA. This result suggests that IAA only moderately affects the germination rate of sugar beet and that the differences in root length between the control and the ACC and IAA treatments, observed in Figures 1–3, are predominantly caused by the hormonal regulation of root growth after germination.

### Ethylene biosynthesis is regulated by auxin during sugar beet seedling growth

**Figure 5:**
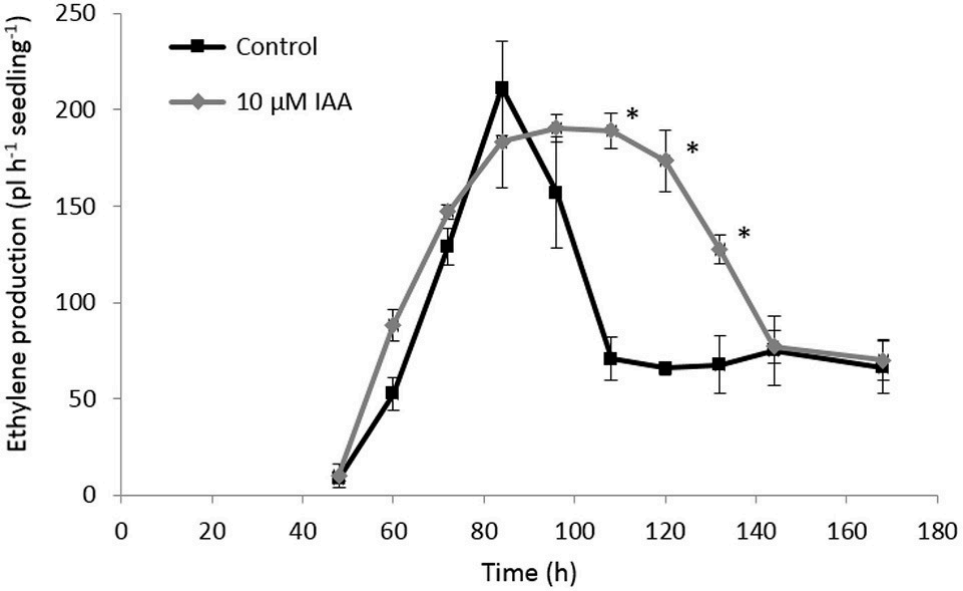
**Effect of indole-3-acetic acid (IAA) on the ethylene production (pL.h^−1^.seedling^−1^) during seedling growth of sugar beet at 20°C in darkness**. Mean values ± *SD* are presented (*n* = 5). The asterisk indicates that the IAA treatment differs significantly from the corresponding controls (*p* < 0.05).

In order to investigate the crosstalk between ethylene and auxin during sugar beet root development, we analyzed the ethylene biosynthesis pathway in sugar beet seedlings treated with IAA. We also measured the content of the ethylene precursor ACC and its derivative MACC, and *ACS* and *ACO* gene expression levels during sugar beet germination and early seedling growth. Our results showed that the onset of ethylene production (48 h after imbibition) was not affected by the IAA treatment (Figure 5). On the other hand, IAA treated seedlings showed higher ethylene production levels compared to the control seedlings at 60 and 72 h after imbibition. The control seedlings reached their maximal ethylene production rate at 84 h after imbibition which subsequently declined again until 108 h after imbibition. Both the IAA treated and control seedlings reached the same maximal ethylene production level, respectively at 72 and 84 h after imbibition. However, the decline in ethylene production for the IAA treated seedlings was slower compared to the control seedlings (between 108 and 132 h after imbibition) resulting in a higher ethylene production rate for the IAA treated seedlings during this period. After 144 h, the ethylene production levels of the control and IAA treatment were the same. This prolonged ethylene production of the IAA treated seedlings might explain the longer root phenotype observed in Figures 1, 2.

**Figure 6:**
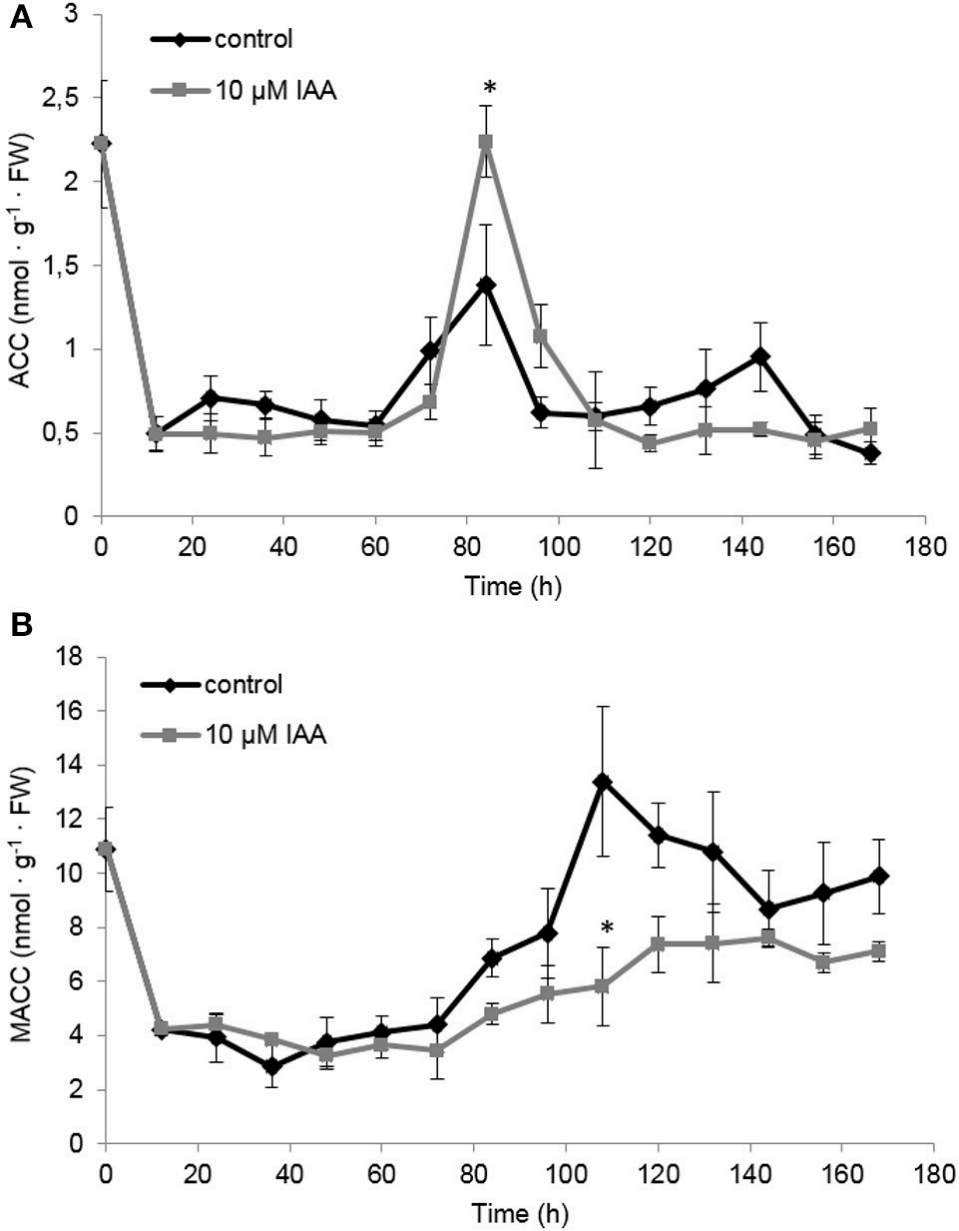
**Effect of indole-3-acetic acid (IAA) on the (A)** 1-aminocyclopropane-1-carboxylic acid (ACC) and **(B)** malonyl-ACC (MACC) profile during seedling growth of sugar beet at 20°C in darkness. Mean values ± *SD* are presented (*n* = 5). The asterisk indicates that the IAA treatment differs significantly from the corresponding controls (*p* < 0.05).

At the metabolic level, the ACC content of the seedlings rapidly decreased during the first 12 h after imbibition (Figure 6A) which is most probably a consequence of water uptake and ACC leaching into the imbibition medium (Hermann et al., 2007; Abts et al., 2014). ACC levels were not significantly affected by IAA during the first 72 h after imbibition. At 84 h after imbibition, ACC levels peaked and the IAA treatment showed a significant higher ACC content compared to the control. The time-point of the ACC peak (84 h) corresponded to the moment when the ethylene production rate was highest (Figure 5).

Similar as ACC, MACC levels were not significantly affected by IAA during the first 72 h after imbibition (Figure 6B). Interestingly, around 84 h MACC levels started to increase and this rise was much more pronounced for the control seedlings compared to the IAA treated seedlings. Untreated seedlings showed a maximal MACC level around 108 h after imbibition followed by a gradual decline. The IAA treatment resulted in a maximal MACC level around 120 h after imbibition after which the levels remained constant. IAA treated seedlings never reach the high MACC levels of untreated seedlings indicating that the formation of MACC is inhibited by the IAA treatment.

**Figure 7:**
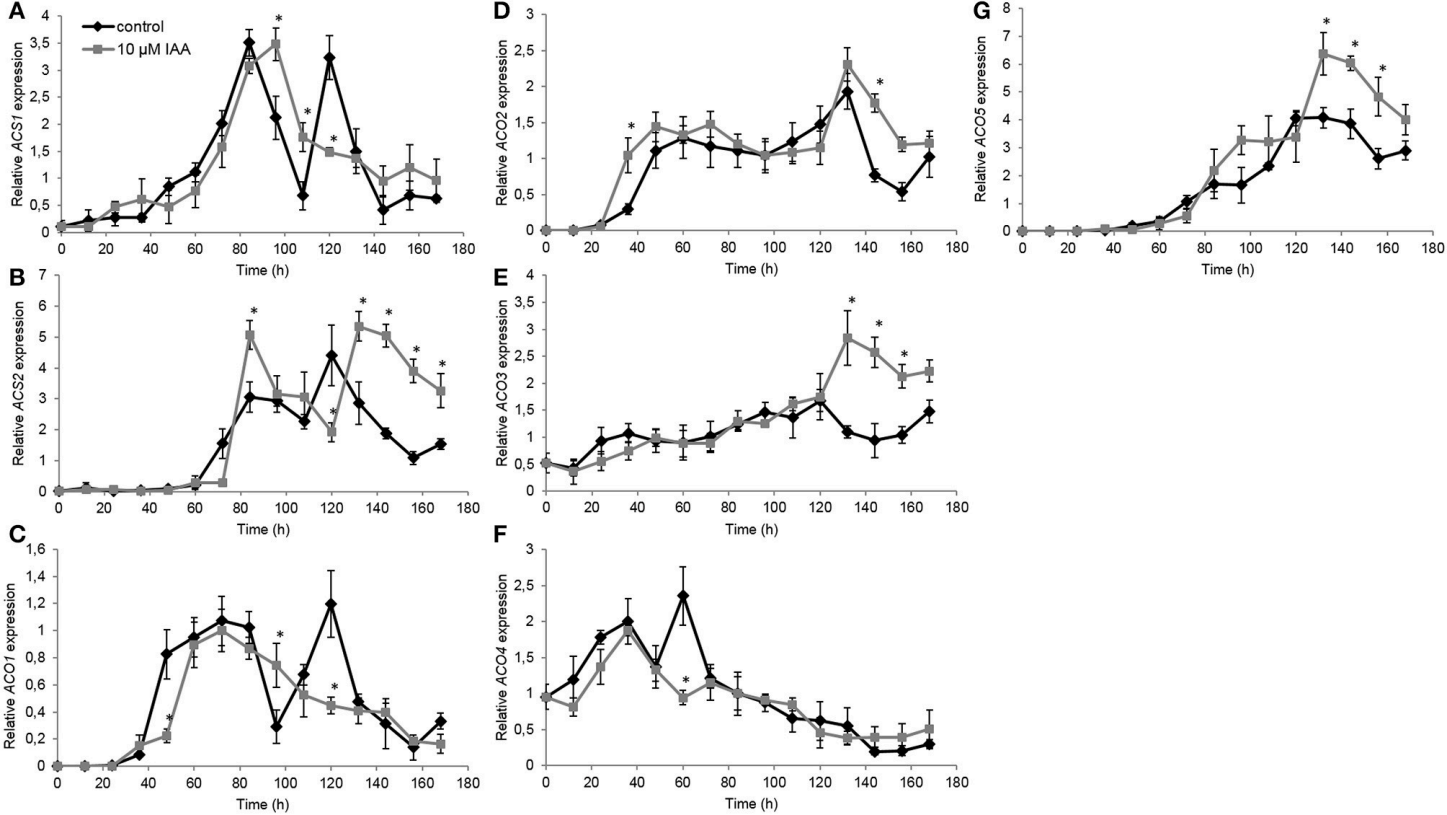
**Effect of indole-3-acetic acid (IAA) on the relative gene expression of *ACS1* (A)**, *ACS2* **(B)**, *ACO1* **(C)**, *ACO2* **(D)**, *ACO3* **(E)**, *ACO4* **(F)**, and *ACO5* **(G)** during seedling growth of sugar beet at 20°C in darkness. Mean values ± *SD* are presented (*n* = 3). The asterisk indicates that the IAA treatment differs significantly from the corresponding controls (*p* < 0.05).

Subsequently we investigated the effect of IAA on the expression of ethylene biosynthesis genes. Therefore, we quantified gene expression levels of all *ACS* and *ACO* isoforms. For *ACS1*, the control treatment showed two time points with peaking transcript levels: the first after 84 h and the second after 120 h (Figure 7A). IAA delayed the first increase with 12 h, while the IAA treatment did not induce a second increase in expression. Similar as *ACS1*, the expression of *ACS2* showed an upregulation after 84 and 120 h in the control treatment (Figure 7B). The IAA treatment resulted in a temporal increase of *ACS2* expression at 84 h. IAA delayed the second increase in transcript levels with ~12 h. Transcript levels of *ACS3* and *ACS4* were not detected at any time point.

The *ACO1* expression profile showed a strong upregulation after 48 h until 96 h (Figure 7C) corresponding with the increase in ethylene production levels during this period (Figure 5). Expression levels quickly dropped and subsequently increased again resulting in a second peak of *ACO1* transcripts around 120 h. IAA delayed the strong upregulation of *ACO1* with 12 h and did not result in a second upregulation of *ACO1* expression. For *ACO2* expression, the IAA treatment resulted in an upregulation after 36 h which was ~12 h earlier compared to the control treatment (Figure 7D). Between 48 and 132 h no differences in *ACO2* transcript levels were observed between the control and the IAA treatment. Thereafter, the IAA treatment showed higher expression levels of *ACO2* compared to the control. The expression profile of *ACO3* was not influenced by IAA until 120 h after the start of imbibition (Figure 7E). Thereafter, IAA showed significantly higher expression levels compared to the control. In general, the expression profile of *ACO4* was not influenced by IAA except from the time point 60 h (Figure 7F). At that time point, the control showed a peak in expression, whereas this increase was lacking in the IAA treatment. Comparable to *ACO3*, transcript levels of *ACO5* were not much changed by IAA until 120 h (Figure 7G). Between 132 and 168 h, the IAA treatment resulted in an increased *ACO5* expression compared to the control which was also observed in the expression pattern of *ACO2* and *ACO3*.

### IAA does not affect ACO activity during sugar beet seedling growth

**Figure 8:**
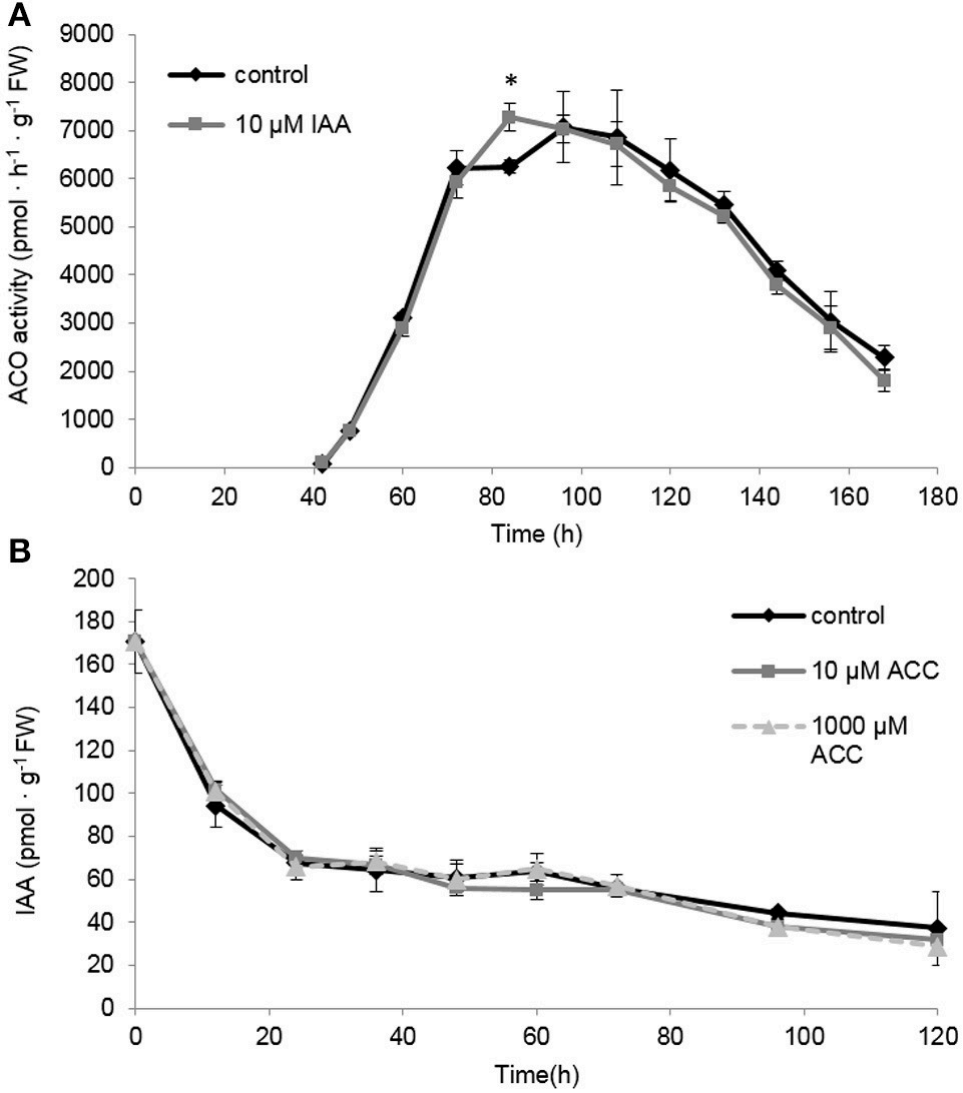
**(A)** The effect of indole-3-acetic acid (IAA) on the *in vivo* ACO enzyme activity during seedling growth of sugar beet at 20°C in darkness. **(B)** Effect of 1-aminocyclopropane-1-carboxylic acid (10 μM ACC, gray solid line; 1,000 μM ACC, gray dotted line) on the indole-3-acetic acid profile (IAA) during seedling growth of sugar beet at 20°C in darkness. Mean values ± *SD* are presented (*n* = 5). The asterisk indicates that the IAA treatment differs significantly from the corresponding controls (*p* < 0.05).

The differences in *ACO* gene expression observed for the IAA treated seedlings (Figures 7C–G) made us wonder if this could explain the sustained ethylene production levels observed in Figure 6. Therefore, we also measured the maximal *in vitro* ACO enzyme activity. We observed that the ACO enzyme activity was not influenced by the IAA treatment, except for a single time point 84 h (Figure 8A). Interestingly, the ACO *in vivo* activity profiles for both the control and the IAA treatment were similar to the ethylene production profile of the IAA treated seedlings (Figure 5). This result indicates that the differences in ethylene production between the control and IAA treatment (Figure 5) are most likely not attributed to a difference in ACO activity (Figure 8A), but rather to a difference in ACC availability.

### Ethylene does not affect IAA levels during sugar beet seedling growth

Because it is well-documented that auxin biosynthesis is also regulated by ethylene during root growth (Ruzicka et al., 2007; Stepanova et al., 2007; Swarup et al., 2007), we investigated the possible effect of ACC on free IAA levels in sugar beet seedlings. In Figure 1 we showed that 10 μM ACC stimulates root elongation while 1 mM ACC inhibits root elongation. However, none of these ACC concentrations influenced the IAA levels compared to the control (Figure 8B). Note that our IAA analyses were conducted on entire seedlings, and that tissue-specific differences in IAA content could be masked. Nonetheless, our results suggest that free IAA levels are not affected by ethylene during seedling development of sugar beet. Figure 8B also shows that endogenous IAA levels drastically drop after imbibition and gradually further decline during seedling development.

## Discussion

Our previous study (Abts et al., 2014) showed that ACC regulates early seedling development of sugar beet in a concentration dependent manner, with low concentrations stimulating root growth and high concentrations inhibiting root growth. However, the regulation of root elongation is often the result of a complex interaction between ethylene and auxin (reviewed by Benková and Hejátko, 2009; Muday et al., 2012). Hence we evaluated the effect of auxin during early root growth of sugar beet and checked for a possible interaction with the ethylene biosynthesis pathway.

### Auxin stimulates root growth in sugar beet by interacting with ethylene

Although auxin is mostly described to inhibit root elongation (Rahman et al., 2007; Ruzicka et al., 2007; Stepanova et al., 2007; Swarup et al., 2007; Alarcón et al., 2012), our results showed that auxin can stimulate root elongation during seedling growth of sugar beet. Evans et al. (1994) also found that low concentrations of auxin can stimulate root elongation in *Arabidopsis*. In a previous study (Abts et al., 2014) we showed that physiological relevant ACC levels also stimulated root elongation in sugar beet in contradiction to many other species where ACC inhibits root elongation. Based on these observations we can conclude that both auxin and ethylene have a stimulating effect on early root growth in sugar beet seedlings.

In this study we also showed that the combined application of auxin and ACC resulted in a cooperative effect with an even longer root. The use of an ethylene and auxin inhibitor (STS and PCIB, respectively) indicated that IAA was not able to overrule the STS effect on root elongation and that PCIB did not affect the stimulating effect of ACC on root elongation. These results suggest that root elongation is primarily regulated by the action of ethylene and not auxin. Additional experiments with other inhibitors of ethylene [e.g., 1-methylcyclopropene (1-MCP), aminooxyacetic acid (AOA), or aminoisobutyric acid (AIB)] and/or auxin [e.g., L-kynurenine, 5-(4-chlorophenyl)-4H-1,2,4-triazole-3-thiol (yucasin), 4-biphenylboronic acid (BBo), 4-phenoxyphenylboronic acid (PPBo)] could be done to further unravel the auxin-ethylene crosstalk in sugar beet root development (Hu et al., 2017). Our results also showed that free IAA levels in the seedling were not affected by the ACC treatment, suggesting that auxin biosynthesis is not stimulated by ethylene in sugar beet in contradiction to previous observations in *Arabidopsis* where an ACC treatment induces IAA production (Ruzicka et al., 2007; Swarup et al., 2007). It should be noted that despite unchanged levels of free IAA, the balance of IAA derivatives and conjugates can regulate the cellular IAA homeostasis and should be assessed in future studies. Altogether, these results indicate that the actual signal resulting in root elongation is likely to originate from ethylene and not from auxin, but it is possible that auxin can still exert an ethylene-independent effect on root elongation in sugar beet.

### Auxin-induced root elongation is achieved by redirecting ACC toward ethylene production instead of MACC, prolonging the total ethylene exposure period

In our previous work we have shown that ethylene production starts just after the radicle emergence of the root during sugar beet germination (Abts et al., 2013). Maximal ethylene levels were observed around completion of germination and subsequently declined during further root growth (Abts et al., 2013). The present study reveals that auxin promotes ethylene production during early stages of root elongation and especially delays the decline in ethylene production ensuring a persisted high rate of ethylene production. This longer exposure period of the seedling toward ethylene might explain why the IAA treatment results in longer roots. These observations also suggest that auxin promotes ethylene biosynthesis during sugar beet seedling development. By dissecting the ethylene biosynthesis pathway we found that the maximal *in vivo* ACO capacity is the same for IAA treated and control seedlings, indicating that the regulation of ethylene biosynthesis is achieved at the level of ACS or ACC itself. It has been stated numerously that ACS is the rate limiting step of ethylene biosynthesis (Yang and Hoffman, 1984). One explanation could be that auxin induces *ACS* expression. It was previously shown in *Arabidopsis* that IAA can specifically and very rapidly induce the expression of *ACS4* in dark grown seedlings (Abel et al., 1995). Although, we did not capture the vary rapid *ACS* responses (within 1 h), our results showed that *ACS* gene expression was not drastically affected by the auxin treatment on the long-term, except during the very late stages of seedling development (later than 132 h after imbibition). The sugar beet *ACS* expression profiles might suggest that the supply of ACC by ACS was more or less similar between IAA treated and untreated seedlings. Nonetheless, we observed a higher ACC level at 84 h after imbibition for the IAA treated seedlings (Figure 6A). Our biochemical analysis of MACC content have revealed that MACC levels in IAA treated seedlings were lower compared to the control seedlings for the entire time frame of the experiment (Figure 6B). This discrepancy in MACC content might suggest that the auxin-induced surplus in ethylene production is likely to be caused by an inhibited conversion of ACC to MACC, leading to a shift in the pool of available ACC toward ethylene. Far too often, the formation of MACC is neglected in ethylene-related crosstalk studies undermining the importance of this ACC derivative, as reflected by our observations in sugar beet.

### Ethylene does not affect the pool of IAA during seedling development of sugar beet

Although, it is generally assumed that ethylene modulates auxin biosynthesis during early root growth (Ruzicka et al., 2007; Stepanova et al., 2007; Swarup et al., 2007; Muday et al., 2012) our study shows that free IAA levels (in the entire seedling) are not affected by the supplementation of ACC during sugar beet seedling development. Hermann et al. (2007) also showed that auxin levels are not altered after an ACC treatment. All combined, this suggests that ethylene has no effect on the level of free IAA in sugar beet. Maybe it is also possible that ethylene affects the level of IAA precursors and/or conjugated forms of IAA. It can also be that ethylene has an effect on IAA signaling or IAA transport, as previously described for *Arabidopsis* (reviewed by Benková and Hejátko, 2009; Muday et al., 2012). Whether this is the case during sugar beet seedling development remains to be investigated.

## Conclusion

Our results have shown that both auxin and ACC can stimulate root elongation in sugar beet seedlings. The combination of IAA and ACC resulted in a cooperative effect on root length but only when supplemented in a low dose. We also demonstrated that externally applied IAA stimulates ethylene production by redirecting the pool of available ACC toward ethylene instead of MACC. The IAA treatment also results in a differential regulation of both *ACS* and *ACO* gene expression during seedling development. All combined this results in a longer and higher ethylene production rate, which in turn stimulates root elongation. On the contrary we did not observe any changes in IAA content during germination when sugar beet seedlings were treated with ACC. We can conclude that auxin stimulates ethylene biosynthesis and not the other way around during sugar beet root development. This suggests that the general ethylene-auxin crosstalk model elucidated in *Arabidopsis* roots (where ethylene promotes auxin production) does not seem to exist in sugar beet.

## Author contributions

WA performed the experiments. WA, BV, and MD designed the experimental work. WA, BV, MD, and BVdP analyzed the data. WA, MD, and BVdP wrote the manuscript.

## Funding

This research and the position of WA was funded by the Department of Biosystems, University of Leuven. BVdP was partially supported by the Belgian American Educational Foundation and the Special Research Fund of the University of Leuven.

### Conflict of interest statement

The authors declare that the research was conducted in the absence of any commercial or financial relationships that could be construed as a potential conflict of interest.

## Supplementary material

Supplementary Figure 1 **Effect of (A)** 10 μM ACC and **(B)** 1,000 μM ACC on the ethylene production (pL.h^−1^.seedling^−1^) during seedling growth of sugar beet at 20°C in darkness. Mean values ± *SD* are presented (*n* = 5).

Supplementary Figure 2 **Effect of different PCIB concentrations (1–100 μM) on root length (mm) of sugar beet seedlings after 6 days at 20°C in darkness**. Mean values ± *SD* are presented (*n* > 10).

Supplementary Figure 3 **Effect of different concentrations of α-(p-chlorophenoxy)isobutyric acid (PCIB) on the ethylene production (pL.h^−1^.seedling^−1^) during seedling growth of sugar beet at 20°C in darkness**. Mean values ± *SD* are presented (*n* = 5). PCIB was dissolved in dimethyl sulfoxide (DMSO) and diluted to the appropriate concentration. The final concentration of DMSO was kept below 0.1% for all treatments.

Supplementary Figure 4 **(A)** Effect of different concentrations of silver thiosulphate (STS) added to the imbibition medium on root length (mm) of sugar beet seedlings after 6 days at 20°C in darkness. Mean values ± *SD* are presented (*n* > 40). Levels of significance (*p* < 0.05) are indicated with the letters a–d. **(B)** Effect of different STS concentrations on the ethylene production (pL.h^−1^.seedling^−1^) during seedling growth of sugar beet at 20°C in darkness. Mean values ± *SD* are presented (*n* = 5).

## References

[B1] Abel S. Nguyen M. D. Chow W. Theologis A. (1995). ASC4, a primary indoleacetic acid-responsive gene encoding 1-aminocyclopropane-1-carboxylate synthase in *Arabidopsis thaliana*. J. Biol. Chem. 270, 19093–19099. 10.1074/jbc.270.32.19093 7642574

[B2] Abts W. Van de Poel B. Vandenbussche B. De Proft M. P. (2014). Ethylene is differentially regulated during sugar beet germination and affects early root growth in a dose-dependent manner. Planta 240, 679–686. 10.1007/s00425-014-2124-0 25034827

[B3] Abts W. Vissers K. Vandenbussche B. De Proft M. P. (2013). Study of ethylene kinetics during and after germination of sugar beet (*Beta vulgaris* L.) seeds and fruits. Seed Sci. Res. 23, 205–210. 10.1017/S0960258513000147

[B4] Alarcón M. V. Lloret P. G. Iglesias D. J. Talón M. Salguero J. (2012). Comparison of growth responses to auxin 1-naphthaleneacetic acid and the ethylene precursor 1-aminocyclopropane-1-carboxylic acid in maize seedling root. Acta Biol. Cracov. 54, 16–23. 10.2478/v10182-012-0001-3

[B5] Atta-Aly M. A. Saltveit M. E. Hobson G. E. (1987). Effect of silver ions on ethylene biosynthesis by tomato tissue. Plant Physiol. 83, 44–48. 10.1104/pp.83.1.44 16665213 PMC1056296

[B6] Benková E. Hejátko J. (2009). Hormone interactions at the root apical meristem. Plant Mol. Biol. 69, 383–396. 10.1007/s11103-008-9393-6 18807199

[B7] Bulens I. Van de Poel B. Hertog M. De Proft M. P. Geeraerd A. H. Nicolaï B. M. (2011). Protocol: an updated integrated methodology for analysis of metabolites and enzyme activities of ethylene biosynthesis. Plant Methods 7, 17–26. 10.1186/1746-4811-7-17 21696643 PMC3142538

[B8] Collett C. E. Harberd N. P. Leyser O. (2000). Hormonal interactions in the control of Arabidopsis hypocotyl elongation. Plant Physiol. 124, 553–562. 10.1104/pp.124.2.553 11027706 PMC59162

[B9] Dohm J. C. Minoche A. E. Holtgräwe D. Capella-Gutiérrez S. Zakrzewski F. Tafer H.. (2014). The genome of the recently domesticated crop plant sugar beet (*Beta vulgaris*). Nature 505, 546–549. 10.1038/nature12817 24352233

[B10] Eliasson L. Bertell G. Bolander E. (1989). Inhibitory action of auxin on root elongation not mediated by ethylene. Plant Physiol. 91, 310–314. 10.1104/pp.91.1.310 16667017 PMC1061992

[B11] Evans M. L. Ishikawa H. Estelle M. A. (1994). Responses of Arabidopsis roots to auxin studied with high temporal resolution: comparison of wild type and auxin-response mutants. Planta 194, 215–222. 10.1007/BF01101680

[B12] Hermann K. Meinhard J. Dobrev P. Linkies A. Pesek B. Hess B.. (2007). 1-Aminocyclopropane-1-carboxylic acid and abscisic acid during the germination of sugar beet (*Beta vulgaris* L.): a comparative study of fruits and seeds. J. Exp. Bot. 58, 3047–3060. 10.1093/jxb/erm162 17761730

[B13] Hoffman N. E. Yang S. F. McKeon T. (1982). Identification and metabolism of 1-(malonylamino)cyclopropane-1-carboxylic acid as a major conjugate of 1-aminocyclopropane-1-carboxylic acid, an ethylene precursor in higher plants. Biochem. Biophys. Res. Commun. 104, 765–770. 10.1016/0006-291X(82)90703-3 7073714

[B14] Hu Y. Vandenbussche F. Van Der Straeten D. (2017). Regulation of seedling growth by ethylene and the ethylene-auxin crosstalk. Planta 245, 467–489. 10.1007/s00425-017-2651-6 28188422

[B15] Lee J. S. Chang W. K. Evans M. L. (1990). Effects of ethylene on the kinetics of curvature and auxin redistribution in gravistimulated roots of *Zea mays*. Plant Physiol. 94, 1770–1775. 10.1104/pp.94.4.1770 11537475 PMC1077451

[B16] Lehman A. Black R. Ecker J. R. (1996). HOOKLESS1, an ethylene response gene, is required for differential cell elongation in Arabidopsis hypocotyls. Cell 85, 183–194. 10.1016/S0092-8674(00)81095-8 8612271

[B17] Lizada M. C. Yang S. F. (1979). Simple and sensitive assay for 1-aminocyclopropane-1-carboxylic acid. Anal. Biochem. 100, 140–145. 10.1016/0003-2697(79)90123-4 543532

[B18] Markakis M. N. De Cnodder T. Lewandowski M. Simon D. Boron A. Balcerowicz D.. (2012). Identification of genes involved in the ACC-mediated control of root cell elongation in *Arabidopsis thaliana*. BMC Plant Biol. 12, 208–218. 10.1186/1471-2229-12-208 23134674 PMC3502322

[B19] Martin M. N. Saftner R. A. (1995). Purification and characterization of 1-aminocyclopropane-1-carboxylic acid N-malonyltransferase from tomato fruit. Plant Physiol. 108, 1241–1249. 10.1104/pp.108.3.1241 12228541 PMC157479

[B20] Muday G. K. Rahman A. Binder B. M. (2012). Auxin and ethylene: collaborators or competitors? Trends Plant Sci. 17, 181–195. 10.1016/j.tplants.2012.02.001 22406007

[B21] Oono Y. Ooura C. Rahman A. Aspuria E. T. Hayashi K. Tanaka A.. (2003). p-Chlorophenoxyisobutyric acid impairs auxin response in Arabidopsis root. Plant Physiol. 133, 1135–1147. 10.1104/pp.103.027847 14526108 PMC281609

[B22] Peck S. C. Kende H. (1995). Sequential induction of the ethylene biosynthesis enzymes by indole-3-acetic acid in etiolated peas. Plant Mol. Biol. 28, 293–301. 10.1007/BF00020248 7599314

[B23] Peck S. C. Kende H. (1998). Differential regulation of genes encoding 1-aminocyclopropane-carboxylate (ACC) synthase in etiolated pea seedlings: effects of indole-3-acetic acid, wounding, and ethylene. Plant Mol. Biol. 38, 977–982. 10.1023/A:1006033030081 9869404

[B24] Pieck M. Yuan Y. Godfrey J. Fisher C. Zolj S. Vaughan D.. (2015). Auxin and tryptophan homeostasis are facilitated by the ISS1/VAS1 aromatic aminotransferase in Arabidopsis. Genetics 201, 185–199. 10.1534/genetics.115.180356 26163189 PMC4566262

[B25] Pierik R. Tholen D. Poorter H. Visser E. J. Voesenek L. A. (2006). The Janus face of ethylene: growth inhibition and stimulation. Trends Plant Sci. 11, 176–183. 10.1016/j.tplants.2006.02.006 16531097

[B26] Pitts R. J. Cernac A. Estelle M. (1998). Auxin and ethylene promote root hair elongation in Arabidopsis. Plant J. 16, 553–560. 10.1046/j.1365-313x.1998.00321.x 10036773

[B27] Polit J. T. Praczyk T. Pernak J. Sobiech L. Jakubiak E. Skrzypczak G. (2014). Inhibition of germination and early growth of rape seed (*Brassica napus* L.) by MCPA in anionic and ester form. Acta Physiol. Plant. 36, 699–711. 10.1007/s11738-013-1448-x

[B28] Prinsen E. van Laer S. Öden S. van Onckelen H. (2000). Auxin analysis, in Methods in Molecular Biology: Plant Hormone Protocols, eds Tucker G. A. Roberts J. A. (Totowa, NJ: Humana Press), 49–65. 10.1385/1-59259-067-5:49 10820736

[B29] Rahman A. Bannigan A. Sulaman W. Pechter P. Blancaflor E. B. Baskin T. I. (2007). Auxin, actin and growth of the *Arabidopsis thaliana* primary root. Plant J. 50, 514–528. 10.1111/j.1365-313X.2007.03068.x 17419848

[B30] Rahman A. Hosokawa S. Oono Y. Amakawa T. Goto N. Tsurumi S. (2002). Auxin and ethylene response interactions during Arabidopsis root hair development dissected by auxin influx modulators. Plant Physiol. 130, 1908–1917. 10.1104/pp.010546 12481073 PMC166701

[B31] Reid M. S. Paul J. L. Farhoomand M. B. Kofranek A. M. Staby G. L. (1980). Pulse treatments with the silver thiosulfate complex extend the vase life of cut carnations. J. Am. Soc. Hortic. Sci. 105, 25–27.

[B32] Rozen S. Skaletsky H. (2000). Primer3 on the WWW for general users and for biologist programmers, in Bioinformatics Methods and Protocols: Methods in Molecular Biology, eds Krawetz S. Misener S. (Totowa, NJ: Humana Press), 365–386. 10.1385/1-59259-192-2:365 10547847

[B33] Ruzicka K. Ljung K. Vanneste S. Podhorská R. Beeckman T. Friml J.. (2007). Ethylene regulates root growth through effects on auxin biosynthesis and transport-dependent auxin distribution. Plant Cell 19, 2197–2212. 10.1105/tpc.107.052126 17630274 PMC1955700

[B34] Stepanova A. N. Yun J. Likhacheva A. V. Alonso J. M. (2007). Multilevel interactions between ethylene and auxin in Arabidopsis roots. Plant Cell 19, 2169–2185. 10.1105/tpc.107.052068 17630276 PMC1955696

[B35] Swarup R. Perry P. Hangenbeek D. Van Der Straeten D. Beemster G. T. Sandberg G.. (2007). Ethylene upregulates auxin biosynthesis in Arabidopsis seedlings to enhance inhibition of root cell elongation. Plant Cell 19, 2186–2196. 10.1105/tpc.107.052100 17630275 PMC1955695

[B36] Tanimoto M. Roberts K. Dolan L. (1995). Ethylene is a positive regulator of root hair development in *Arabidopsis thaliana*. Plant J. 8, 943–948. 10.1046/j.1365-313X.1995.8060943.x 8580964

[B37] Tsuchisaka A. Theologis A. (2004). Unique and overlapping expression patterns among the Arabidopsis 1-aminocyclopropane-1-carboxylate synthase gene family members. Plant Physiol. 136, 2982–3000. 10.1104/pp.104.049999 15466221 PMC523360

[B38] Van de Poel B. Smet D. Van Der Straeten D. (2015). Ethylene and hormonal crosstalk in vegetative growth and development. Plant Physiol. 169, 61–72. 10.1104/pp.15.00724 26232489 PMC4577414

[B39] Van de Poel B. Van Der Straeten D. (2014). 1-Aminocyclopropane-1-carboxylic acid (ACC) in plants: more than just the precursor of ethylene! Front. Plant Sci. 5:640. 10.3389/fpls.2014.00640 25426135 PMC4227472

[B40] Yang S. F. Hoffman N. E. (1984). Ethylene biosynthesis and its regulation higher plants. Annu. Rev. Plant Physiol. 35, 155–189. 10.1146/annurev.pp.35.060184.001103

[B41] Zheng Z. Guo Y. Novak O. Dai Z. Zhao Y. Ljung K.. (2013). Coordination of auxin and ethylene biosynthesis by the aminotransferase VAS1. Nat. Chem. Biol. 9, 244–246. 10.1038/nchembio.1178 23377040 PMC3948326

