# Supplementary figures and images for: The Role of Auxin-Ethylene Crosstalk in Orchestrating Primary Root Elongation in Sugar Beet
Source: Front Plant Sci. 2017 Mar 30;8:444. doi: 10.3389/fpls.2017.00444 (PMC5371662; doi:10.3389/fpls.2017.00444)

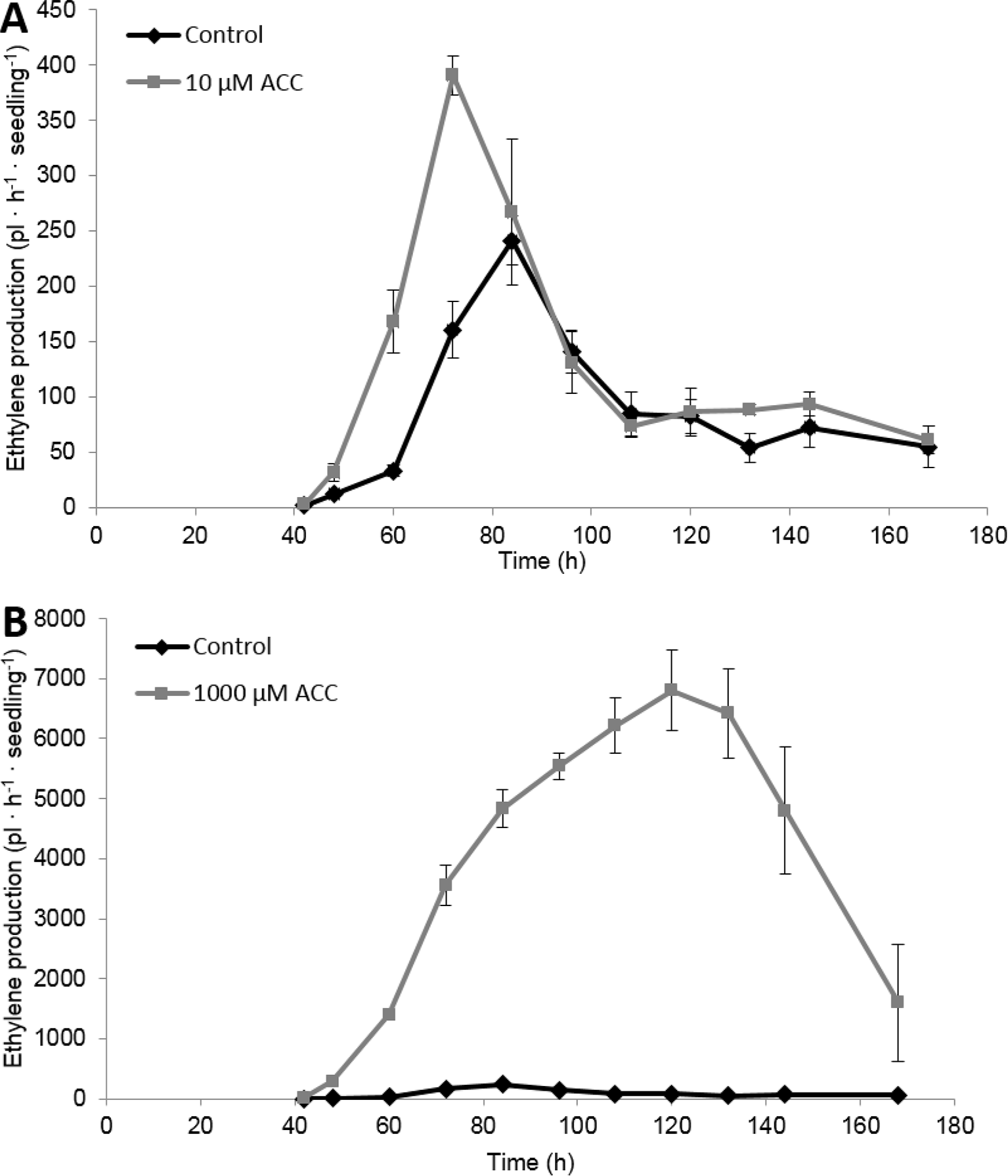

Supplement: Supplementary Figure 1 — Effect of (A) 10 μM ACC and (B) 1,000 μM ACC on the ethylene production (pL.h−1.seedling−1) during seedling growth of sugar beet at 20°C in darkness. Mean values ± SD are presented (n = 5). [file Image1.TIF]

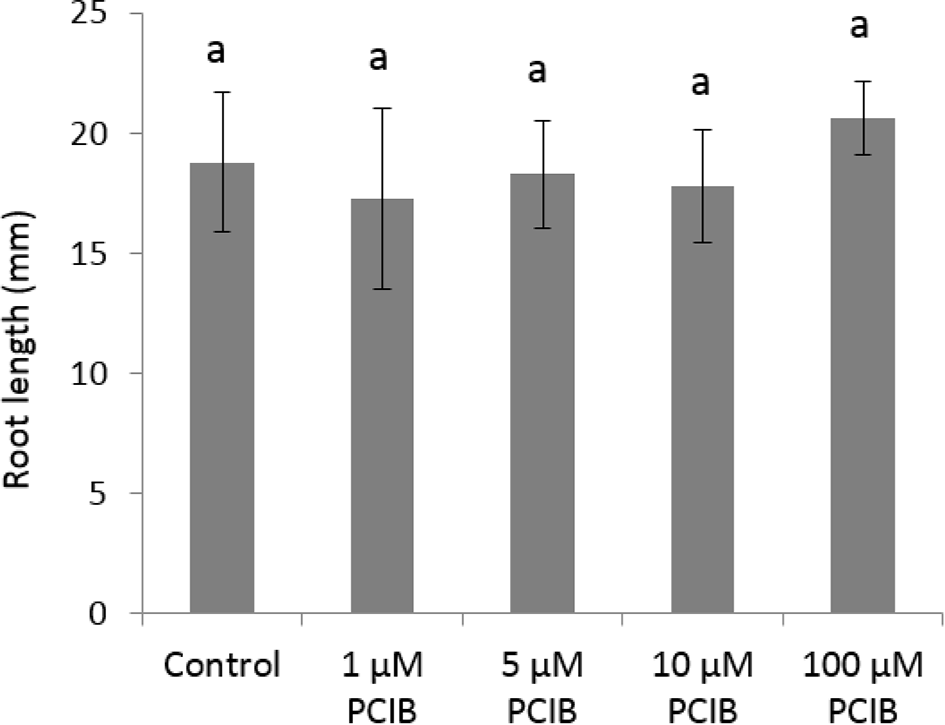

Supplement: Supplementary Figure 2 — Effect of different PCIB concentrations (1–100 μM) on root length (mm) of sugar beet seedlings after 6 days at 20°C in darkness. Mean values ± SD are presented (n > 10). [file Image2.TIF]

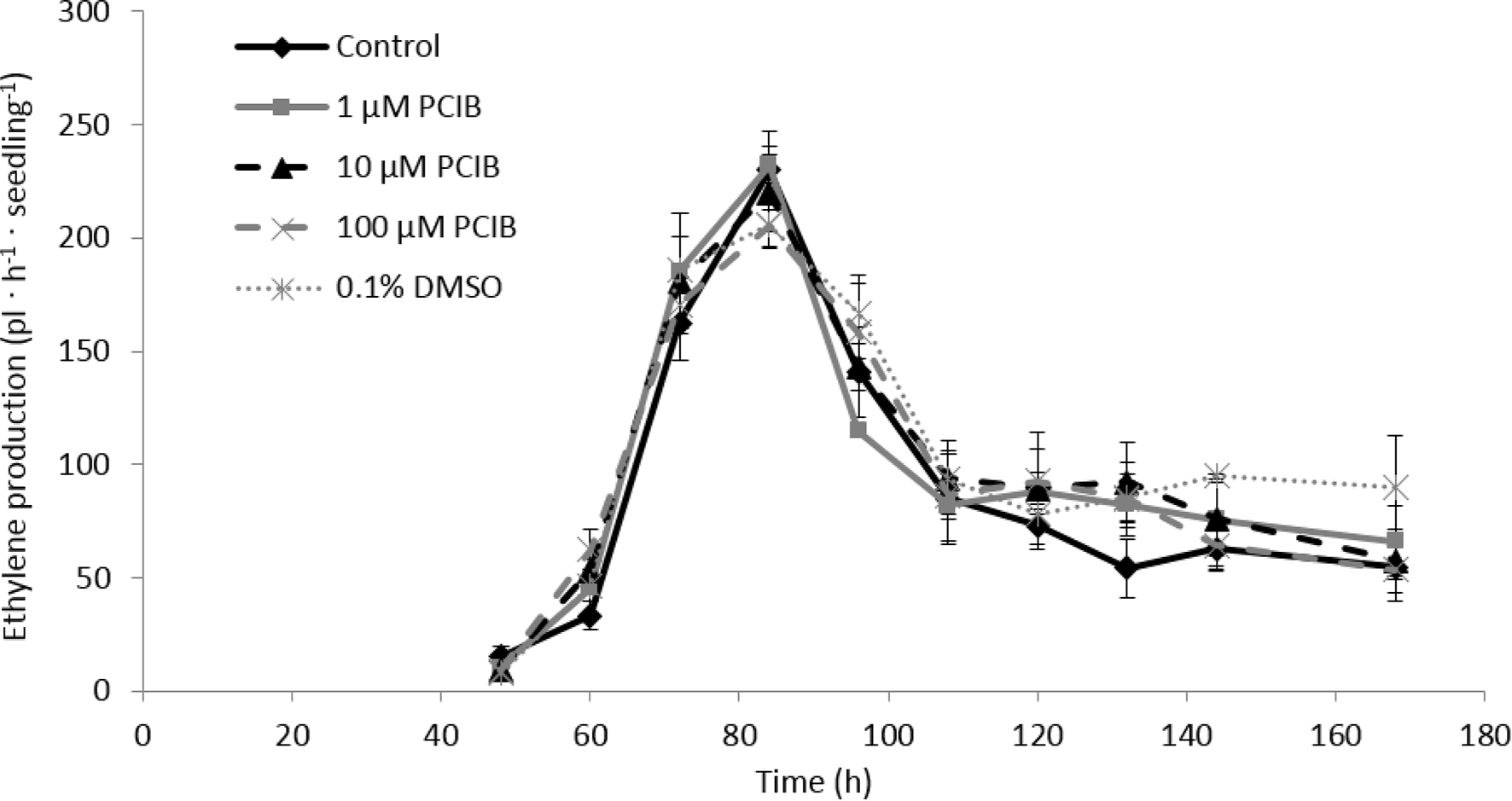

Supplement: Supplementary Figure 3 — Effect of different concentrations of α-(p-chlorophenoxy)isobutyric acid (PCIB) on the ethylene production (pL.h−1.seedling−1) during seedling growth of sugar beet at 20°C in darkness. Mean values ± SD are presented (n = 5). PCIB was dissolved in dimethyl sulfoxide (DMSO) and diluted to the appropriate concentration. The final concentration of DMSO was kept below 0.1% for all treatments. [file Image3.TIF]

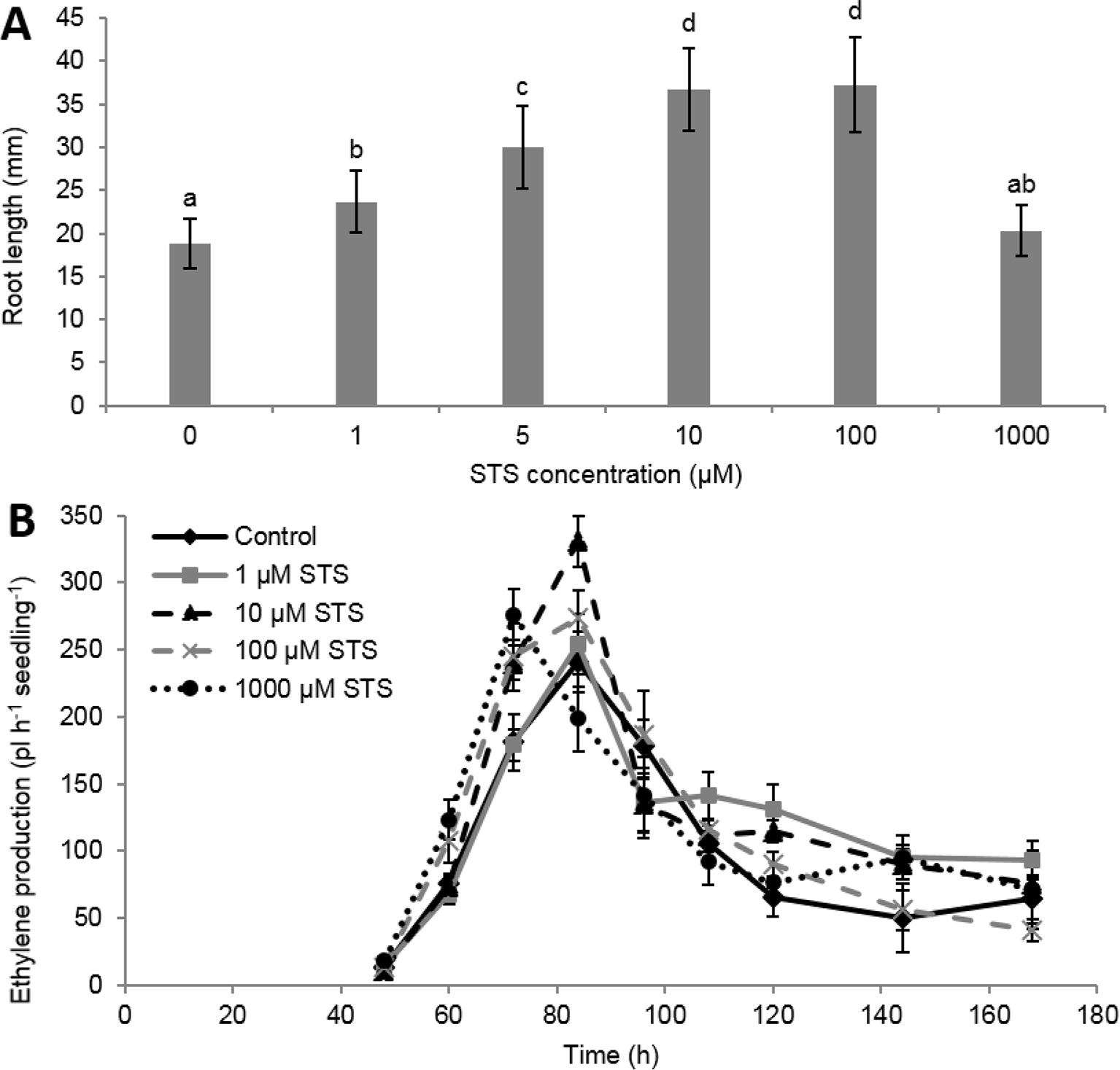

Supplement: Supplementary Figure 4 — (A) Effect of different concentrations of silver thiosulphate (STS) added to the imbibition medium on root length (mm) of sugar beet seedlings after 6 days at 20°C in darkness. Mean values ± SD are presented (n > 40). Levels of significance (p < 0.05) are indicated with the letters a–d. (B) Effect of different STS concentrations on the ethylene production (pL.h−1.seedling−1) during seedling growth of sugar beet at 20°C in darkness. Mean values ± SD are presented (n = 5). [file Image4.TIF]
